# Harnessing machine learning for development of microbiome therapeutics

**DOI:** 10.1080/19490976.2021.1872323

**Published:** 2021-01-30

**Authors:** Laura E. McCoubrey, Moe Elbadawi, Mine Orlu, Simon Gaisford, Abdul W. Basit

**Affiliations:** a UCL School of Pharmacy, University College London, London, UK; b FabRx Ltd., Ashford, Kent, UK

**Keywords:** microbiome, machine learning, artificial intelligence, drug product development, microbial therapeutics, clinical translation, pharmaceutical sciences, COVID-19, colonic drug delivery, personalized medicines

## Abstract

The last twenty years of seminal microbiome research has uncovered microbiota’s intrinsic relationship with human health. Studies elucidating the relationship between an unbalanced microbiome and disease are currently published daily. As such, microbiome big data have become a reality that provide a mine of information for the development of new therapeutics. Machine learning (ML), a branch of artificial intelligence, offers powerful techniques for big data analysis and prediction-making, that are out of reach of human intellect alone. This review will explore how ML can be applied for the development of microbiome-targeted therapeutics. A background on ML will be given, followed by a guide on where to find reliable microbiome big data. Existing applications and opportunities will be discussed, including the use of ML to discover, design, and characterize microbiome therapeutics. The use of ML to optimize advanced processes, such as 3D printing and *in silico* prediction of drug-microbiome interactions, will also be highlighted. Finally, barriers to adoption of ML in academic and industrial settings will be examined, concluded by a future outlook for the field.

## Introduction

The human microbiome describes the genomes of trillions of microorganisms that live on and within the human body. Members of the microbiota include bacteria, viruses, fungi, archaea, and protozoa; collectively they inhabit nearly every imaginable region of their host.^1^ Bacteria alone encode for over 100-fold more unique genes than humans.^2^ Subsequently, microbiota is known to exert extensive influence upon human health and metabolism. For example, the development of metabolic syndrome, Parkinson’s disease, inflammatory bowel disease, and periodontitis are strongly associated with imbalanced, also known as ‘dysbiotic’, microbiome compositions.^3–6^ With regards to metabolism, the microbiome wields impactful metabolic capacity. The gut microbiome plays a crucial role in the degradation of dietary fibers, proteins, polyphenols, and starches which would be otherwise indigestible by their human host.^7,8^ Endogenous moieties broken down by microbiota include bile acids, hormones, and intestinal mucus.^9^ Drugs are also highly susceptible to metabolism by microbiota. A seminal study found that of 271 commonly administered oral drugs, 176 (64.9%) of them were significantly metabolized by at least one strain of 76 human gut bacteria.^10^ Due to substantial differences between individuals’ microbiomes, drug metabolism by microbiota can result in significant inter-patient variability in pharmacokinetics and pharmacodynamics.^11^

**Figure 1.**
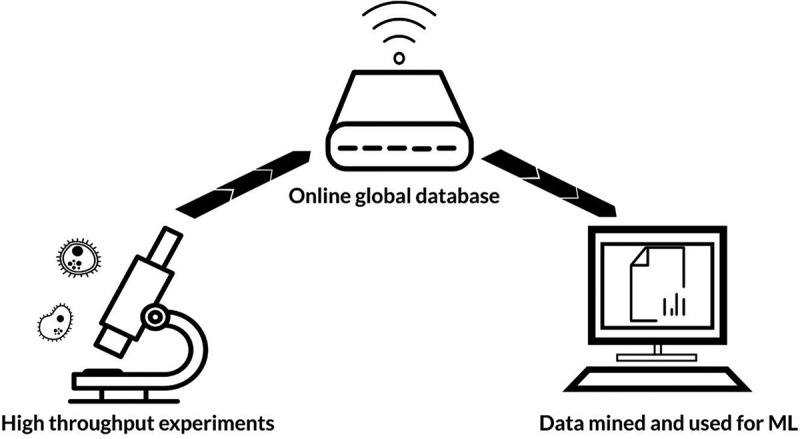
Flow of information for machine learning (ML). Vast quantities of experimental data are uploaded to online globally accessible databases. These data can then be mined and used by ML algorithms

From the beginning of the 21^st^ century, microbiome-based research has amassed at a progressive rate. In the mid-2000s, affordable and accurate DNA sequencing methods facilitated the commencement of the Human Microbiome Project: a multi-site collaboration leading to the genomic profiling of microbiota at key body sites.^12^ Later, the project assessed the role of the microbiome in human health and disease.^13^ In parallel to this field-leading work, many other laboratories worldwide have contributed to the now extensive knowledge base concerning the microbiome. Vast quantities of data have been made globally accessible through publications and online databases, such as the NIH Human Microbiome Project Data Portal; MicrobiomeDB; and China National GeneBank.^14–16^ Such data are being increasingly utilized by scientists to design microbiome-targeted therapies and predict microbiome-drug interactions. Due to the scale of available data, artificial intelligence (AI) offers a means to quickly and accurately mine, process, and analyze available information. Whilst it may take a human years to identify patterns in terabytes of data, AI algorithms can efficiently perform operations within seconds to minutes, depending on computer processing power, data size, and algorithm complexity. There are four main types of AI: machine learning (ML); natural language processing; rule-based expert systems; and robotics.^17^ By far, ML is the most extensively used AI technique within the microbiome field.^18–20^ ML uses coded computer algorithms to identify patterns in data, which facilitates the classification of new information or prediction of future outcomes (Figure 1).

This review will provide an accessible introduction to ML and highlight its current applications within the microbiome therapeutics field; namely, using ML as a tool for designing microbiome-targeted therapies, and predicting drug-microbiome interactions. An overview of useful databases for ML microbiome projects will also be presented. Opportunities for utilizing ML will be addressed, with a description of barriers to adoption in academic and industrial settings. Finally, an outlook for the field will be described.

## Machine Learning Methods

The origins of ML date back to the 1600s, when the first mechanical calculator, and the modern binary system were created. These inventions, still integral to contemporary ML, were translated to modern ML by the mid-1950s. In 1952, IBM’s Arthur Samuel coined the phrase ‘machine learning’ upon creating a computer program that could improve its skill in the game of checkers the more it played. Later, in 1997, IBM’s Deep Blue computer rose to fame after successfully beating the chess grandmaster Garry Kasparov. Though Kasparov requested a rematch, Deep Blue was retired with its clean slate without encore. At the turn of the millennium, ML algorithms were progressively applied to tasks other than winning board games. Research on ML began hitting an exponential rate; algorithms were developed to detect cancer more accurately than radiologists, identify human faces from images, predict consumer behavior, speak like a native, and lipread, to name just a few.^21^ Today, ML technology has been applied to almost every sector on a global scale. Still ML capabilities are advancing at a rapid rate; new research articles are published daily that solve previously impenetrable challenges. Healthcare has especially benefitted from ML; algorithms are now available to diagnose disease, develop drugs, recommend personalized treatment plans, and predict future health outcomes. Though it will take some time for widespread clinical adoption, ML promises a future of consistent, probability, and evidence-based medicine.^17^

ML can be split into two parts: supervised and unsupervised learning.^18^ Supervised learning involves directing an algorithm to solve a pre-defined problem. For example, ‘will this newly synthesized drug be metabolized by gut microbiota?’ To generate an answer, the ML software must have access to data that have been labelled. To pertain to the previous example, the ML algorithm would be supplied with a list of drugs, and their molecular features, labeled as being susceptible or not susceptible to gut microbiota metabolism. The algorithm would then look for similarities between the newly synthesized drug, and the list of labeled drugs, by examining their molecular features. A prediction, based on probability, would then be outputted based on whether the new drug has more in common with drugs that are known to be metabolized, or those that are known to remain intact. This is known as a classification task. The ML model can then be validated by performing experiments testing microbiome-drug metabolism in a laboratory setting.

**Figure 2.**
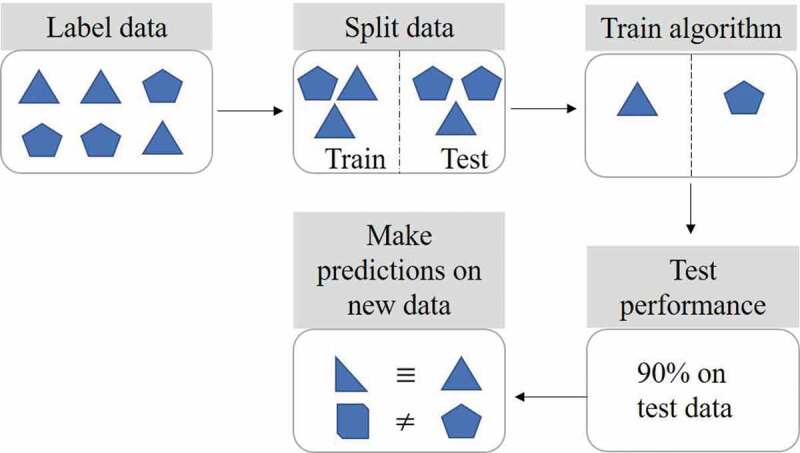
Supervised machine learning steps. Labeled data is split into training and testing sets. The algorithm is then trained to learn the differences between the labeled data. The test set is used to check and refine algorithm performance. Predictions can subsequently be made using new data previously unseen by the algorithm

An entire dataset can be used to train an ML model, however, it is a common practice to split data into separate training and testing sets. The latter is used to test the model’s performance on new and unseen data, if obtaining new data is not readily attainable. The training set can be further split into training and validation sets, wherein the latter is used to further refine the model. Supervised algorithms can either classify new data, e.g. ‘metabolized’ or ‘not metabolized’, or they can predict a numerical value, such as ‘percentage of dose metabolized’. The output of the algorithm (classification or numerical value) depends on how the dataset is labeled, and whether a classification or regression analysis is performed. Several types of supervised ML algorithms exist, these include basic linear methods, decision trees, support vector machines, and advanced neural networks (Figure 2).

Compared to supervised methods, unsupervised learning does not address any pre-defined questions.^22^ At all stages of data mining and ML, the chance of bias should be reduced as much as possible. One could say that introducing a question to an algorithm leads to bias, as the algorithm will look to solve that particular problem. In unsupervised learning, an ML algorithm works to identify patterns in data without any prior operator input. This can subsequently lead to elements being identified that could not be conceived by the operator. Unsupervised ML methods can produce clustering or association outputs. Clustering algorithms identify distinct groups within data; association algorithms output rules found within data. Common unsupervised ML techniques include k-means clustering, principal component analysis, and k nearest neighbors.

At the intersection between supervised and unsupervised learning is semi-supervised ML.^18^ Semi-supervised learning involves using a partly labeled set of data. The ML algorithm uses unsupervised learning to label the unlabeled data, by drawing conclusions from the labeled data. Following that, supervised techniques can be employed to answer defined questions about the labeled information. In practice semi-supervised learning is useful, as real-world datasets are often incompletely labeled due to poor formatting at the time of conception. Hand-labeling data can be extremely time-consuming, error-prone, and expensive, especially if datasets are particularly large and complex. The automated labeling step in semi-supervised learning provides a quick and unbiased way to format previously difficult data.

The final type of ML, reinforcement learning, is similar to supervised learning in the sense that it is goal-directed.^23^ Though unlike supervised learning, reinforcement learning does not require labeled data. Instead, it is supplied with a set of rules around a problem. For example, an operator may input ‘if a new formulation protects a drug from microbiome metabolism, this is a positive action. If the drug is degraded by the microbiota, this is a negative action’. Reinforcement learning solves pre-defined problems in a self-teaching, iterative manner. An algorithm will carry out actions to attempt to solve a problem; the closer an action gets to a solution, the more reward experienced in the system. If a given action results in an outcome that veers away from a solution, then the system will experience punishment. Over time, the algorithm will learn what types of actions are rewarding. In this way, a path toward problem solution is paved. In order to build an optimum path to problem solution, reinforcement learning algorithms face the dilemma of attempting new actions whilst concurrently aiming to maximize reward, this is known as the exploration vs. exploitation trade-off.^24^ Reinforcement learning is probably best known for its use in the mastery of games, such as the incredibly complex and ancient board game ‘Go’. In 2017, reinforcement learning reached superhuman performance within Go, without any input of data: the algorithm was its own teacher.^25^ Common types of reinforcement learning include temporal-difference learning, Q-learning, and state-action-reward-state-action (SARSA) learning (Table 1).^24^

**Table 1.** Types of machine learning

ML Type	Defined question asked?	Is data labeled?	Outputs	Example Algorithms
Supervised	Yes	Yes (fully)	Classification Real values	Linear regression Logistic regression Decision trees Neural networks
Unsupervised	No	No	Clustering Association	K-means clustering Principal component analysis K-nearest neighbors
Semi-supervised	Yes	Yes (partly)	Classification Real values Clustering Association	Any supervised or unsupervised method
Reinforcement	Yes	No (rules supplied instead)	Actions moving toward a solution	Temporal-difference Q-learning SARSA

**Figure 3.**
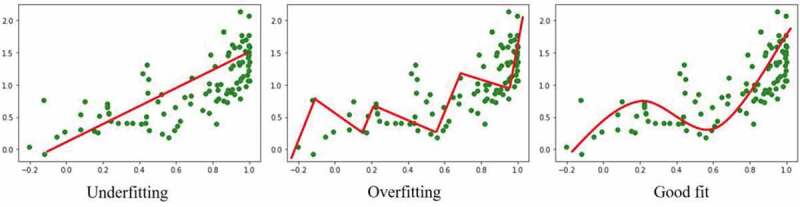
Illustration of underfitting and overfitting in simple regression machine learning. Data points are represented by green markers and model fitting by a red line

In all types of ML, it is important for operators to be aware of over or under fitting algorithms. Overfitting occurs when an algorithm is too sensitive to patterns in training data, leading to perfect accuracy when training, but inadequate performance during testing and validation. On the other hand, if an algorithm is not sensitive enough, and too simple, then it will not be able to model the training data and is expected to perform poorly when generalized to the testing dataset. Finding an optimal balance between algorithm underfitting and overfitting is a crucial component of ML engineering. Inadequate performance of ML algorithms is typically due to poor fitting; it can take a great deal of ML engineers’ time to tweak and perfect ML parameters to find a sweet spot (Figure 3).^26^

## Databases For Ml Microbiome Projects

### What makes a good database?

The quality of data used for ML projects is paramount. Weak, invalid data will lead to weak and invalid ML outputs: ‘garbage in equals garbage out’. Whilst large datasets are favorable for ML projects, as more information invariably leads to higher internal and external validity, big data often come with challenges.^27^ Firstly, it is important to validate the source of data. Within microbiome science, it is common for researchers to use individuals’ stools to sample their gut microbiomes, however it is becoming increasingly clear that stools may not be the most accurate method for profiling the gut microbiome.^28^ Though assaying of stools is noninvasive and cost-effective, microbiota composition changes across the intestinal tract, therefore stools cannot provide a snapshot of any one gastrointestinal niche.^29,30^ Many reputable databases contain stool microbiome data, and the information should not be ignored as it can provide useful insights.^14,31–33^ It is, however, important to be aware of sampling methodology when conducting ML analysis to allow for valid interpretation. Another detail to investigate when examining a microbiome dataset is the population sampled. Microbiome composition is well known to differ with respect to a plethora of factors, including age, sex, health, diet, ethnicity, diet, and drug use.^34–39^ If a ML project aims to draw universally valid interpretations, scientists must consider how globally representative the input data is.

The amount of data available in databases is another important factor. Generally, the more data available to an ML project the better, as the algorithm will naturally be exposed to more information with more variance, allowing increased prediction accuracies.^40^ However, the impact database size has on ML predictions does vary considerably based on the problem being analyzed, and the complexity of the algorithm. For example, if there is a very strong pattern in data, such as ‘ionized drugs are more water soluble than neutral drugs’, less data are required to teach an ML algorithm. Furthermore, some algorithms inherently require larger amounts of data, due to their intricacy and complexity. Simpler algorithms such as linear regression and basic decision trees typically need less data compared to neural networks and deep learning algorithms with multiple layers.^41^ Unfortunately, there is no single minimum amount of data needed for all types of ML. A decision on appropriate sample size can be reached using several methods, such as acquiring expert opinion, operator experience, and observing sample sizes in other similar published projects. For a given project, a plot of ‘dataset size vs. model prediction performance’ can be constructed using a subset of available data. This will allow reasonable projection of potential amounts of data required for a given performance score.^42^ Though insufficient dataset size is a common problem in the general field of ML, big data have become intrinsic to microbiome research. DNA sequencing, metabolomics, proteomics, and *in silico* representation of drugs produce enormous quantities of data that describe the minutiae of the microbiota-host relationship.^43^ Though vast quantities of data can help to build highly predictive ML models, very large datasets (reaching terabytes) do come with problems.^44^ For one, it is not guaranteed that big data are entirely without bias and fully globally representative. Populations less likely to be involved in microbiome research should not be disregarded; thus, it is important to always examine the source of data, no matter its scale. Next, data in repositories are commonly ‘messy’, containing missing values, unequal scaling, and background noise from poorly performed experiments or incorrect data labeling.^45,46^ Clearly, without rectification messy data will adversely impact the predictive power of ML algorithms. Data cleaning is a key part of ML projects. Before any algorithm training or testing begins, it is imperative that messy data are pre-processed. Contrary to common belief, machine learning engineers do not spend most of their time building, training, and refining algorithms. A 2020 study found that data pre-processing (cleaning, labeling, compiling) is on average allocated over 75% of ML project time.^47^ There are a myriad of techniques applied to data pre-processing, and full description of these is outside the scope of this article. A couple of existing sources summarizes this topic well.^22,48^ In brief, missing values can be removed or imputed with best estimates.^49^ Units of data features, such as drug molecular weight or LogP, will be transformed to a similar scale, to avoid giving undue weight to smaller units (e.g. 100 mg may be treated as more important than 0.1 g by an algorithm).^50^ Outliers in datasets may also be removed if they are judged as anomalies.

### Useful databases

Projects utilizing ML within the field of microbiome therapeutics will likely require information on drugs, microbiota, and microbiota-human interactions. With a few exceptions, large databases bringing this information together do not yet exist, therefore it is necessary to mine data from multiple sources. Table 2 gives a summary of some reputable databases with potential utility for ML projects in this field.

**Table 2.** Summary of key databases with potential utility in ML microbiome projects. Databases have been divided into: drugs, drug-microbiota interactions, microbiota, and microbiota-human interactions

Database	Description	URL
*Drugs*		
BindingDB	A binding affinity database focusing on drug-protein interactions.	http://www.bindingdb.org/bind/index.jsp
ChEMBL	A manually curated database of 2 million bioactive molecules bringing together chemical, activity, and genomic data.	https://www.ebi.ac.uk/chembl/
ChemDB	A user-friendly database incorporating over 5 million small molecules annotated with important chemical features, e.g. predicted solubility.	http://cdb.ics.uci.edu/
ChemSpider	A chemical structure database providing information on over 67 million entities from hundreds of sources.	http://www.chemspider.com/
DrugBank	A bioinformatics and cheminformatics database providing information on over 13,500 drug entities, including biologics.	https://www.drugbank.ca/
Open Babel	A wiki-led chemical toolbox suitable for use in many ML programs.	http://openbabel.org/wiki/Main_Page
PharmGKB	A smaller database of 708 drugs annotated with information on how genetic variation can impact clinical response.	https://www.pharmgkb.org/
RDKit	A cheminformatics software for use in ML projects in Python.	https://www.rdkit.org/
Super Natural	Database of over 325,000 natural products providing information on physicochemical properties and toxicity.	http://bioinf-applied.charite.de/supernatural_new/index.php?site=home
*Drug-microbiota interactions*		
DrugBug	Database constructed using ML to predict the metabolism of drugs by bacteria found in the human gut.^51^	http://metagenomics.iiserb.ac.in/drugbug/
*Microbiota*		
BacDive	Database providing taxonomic, physiological, and environmental data on over 80,000 strains of bacteria.	https://bacdive.dsmz.de/
EnsemblBacteria	Database for bacterial and archaeal genomes.	http://bacteria.ensembl.org/index.html
European Nucleotide Archive	Database providing comprehensive nucleotide sequencing information for bacteria, viruses, and fungi, protozoa and archaea.	https://www.ebi.ac.uk/ena/browser/home
Gold	Database providing genomic information for eukaryotes, prokaryotes, and viruses.	https://gold.jgi.doe.gov/
IMG/M	Metagenomic database for microbiota, annotated with functions.	https://img.jgi.doe.gov/
NCBI Microbial Genomes	Comprehensive database covering information on microbial genomes, functions, and recent corresponding publications.	https://www.ncbi.nlm.nih.gov/genome/microbes/
*Microbiota-human interactions*		
Disbiome	Database covering microbiota changes in disease states.	https://disbiome.ugent.be/home
eHOMD	Expanded Human Oral Microbiome Database: genomic data on 775 microbial species in the upper GI and respiratory tracts.	http://www.homd.org/
HMDB	The human metabolome database containing information on over 114,000 human metabolites.	https://hmdb.ca/
Human Microbiome Project	Genomic characterization of microbiota at five body sites (HMP1), and information on microbiota-human interactions in disease (iHMP).	https://www.hmpdacc.org/
MDB	Microbiome database from China National GeneBank, providing metadata on both host-associated (humans, rodents, pigs) and environmental microbes.	https://db.cngb.org/microbiome/
MGnify	Database providing genomic, proteomic, and metabolomic data on human and environmental biomes.	https://www.ebi.ac.uk/metagenomics/
MicrobiomeDB	Data mining platform for microbiome experiments.	https://microbiomedb.org/mbio/app/
UniProt	Detailed database providing the proteomes of almost 200,000 microbiota and humans.	https://www.uniprot.org/
Virtual Metabolic Human	Database on human and gut microbial metabolism with corresponding disease and nutritional information.	https://www.vmh.life/#home

## Applications And Opportunities

### Design and discovery of microbiome therapeutics

The last few years have seen an upsurge in publications utilizing ML within microbiome research, though ML used to specifically design new therapeutics targeted to the microbiome is still in its infancy. The expansion of microbiome ML work naturally follows the curation of large accessible databases.^14–16^ With the elucidation of host-microbiota metabolomics, proteomics, and genomics, disease phenotypes can be identified.^52–56^ Whilst scouring research literature and databases for microbiome disease markers could be time-consuming for a human, ML algorithms output information much more efficiently. Once it is known what elements of the microbiome cause disease, it is possible to begin work toward preventative agents and treatments. Dysbiosis at various body sites has been associated with many diseases. For example, perturbations in the proportions of phyla Firmicutes and Bacteroidetes in the gut are strongly linked with several pathologies, including ulcerative colitis, obesity, and motor neuron disease.^57–59^ Etiological factors are believed to be increased intestinal permeability and inflammation, decreased short-chain fatty acid (SCFA) production, and increased levels of lipoprotein lipase.^3,60^ A variety of ML methods, such as support vector machines, neural networks, and logistic regression, have been utilized to identify microbiota features present in several disease states.^61^ Gupta et al. have developed a classification ML model that can predict individuals’ health status based on microbiome profiling.^62^ Elsewhere, human-bacteria protein-protein interactions have been successfully predicted.^63^ A study by Zeevi et al. used an ML algorithm integrating 800 people’s blood parameters, dietary habits, anthropometrics, physical activity, and gut microbiome profile to predict postprandial glycemic response.^64^ Such work illustrates how precision microbiome medicine can become a reality.^65^ The promise for ML-led microbiome precision medicine within oncology has been well described by Cammarota et al.^26^

**Figure 4.**
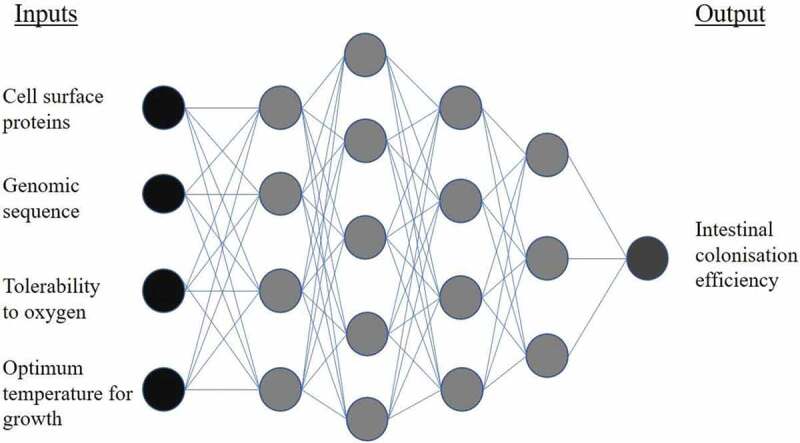
Example of a deep neural network for application in probiotic screening. Input information describing a bacterial strain is fed into hidden layers of the network. Progressing through the layers, the algorithm approaches its output: the predicted intestinal colonization efficiency of the bacteria, when administered as a probiotic

Drug discovery scientists have utilized ML for years to identify novel molecules with therapeutic potential.^41,66–68^ Supervised, unsupervised, semi-supervised, and reinforcement learning styles have all been applied to novel drug-target predictions: methods which could be easily employed for identification of microbiome therapeutics.^69–72^ ML has also been applied to drug repurposing, allowing screening of thousands of marketed drugs for alternative therapeutic purposes.^73^ In 2018 over 1,000 marketed drugs were screened for activity against 40 gut bacteria strains: 24% of the drugs were found to inhibit growth of at least one strain, despite the majority not being marketed for antibiotic purposes.^35^ This highlights the potential for repurposing within microbiome medicine. Prebiotics, entities that promote the growth of beneficial microbiota, are popular and efficacious interventions within microbiome medicine.^74–76^ A recent study using both supervised and unsupervised ML has elucidated and quantified the importance of the structure-property relationships of beta-glucans as prebiotics.^77^ Probiotics, live microorganisms that when administered in adequate amounts confer a health benefit, are commonly investigated microbiome therapeutics.^78^ Currently identification of probiotic candidates usually occurs via a top-down approach, whereby bacteria are selected based on the observation that they are enriched in healthy individuals.^65^ Though this methodology has discovered useful probiotics such as *Bifidobacterium, Lactobacillus*, and *Akkermansia muciniphila*, it gives little consideration for therapeutic mechanism, dose, or heterogeneity between individuals.^60,79^ A bottom-up approach could exploit ML techniques to identify mechanisms of probiotic action, and search for microbiota that fulfils these actions.^65^ Algorithms could consider a multitude of factors before recommending a probiotic, such as efficacy, bioavailability, risk of toxicity, ease of formulation, and scalability. Deep learning techniques, which can incorporate multiple layers of ML algorithms, would be well suited to solve these kinds of complex tasks (Figure 4).^41^

### Formulation of microbiome therapeutics

Once active moieties have been selected, then pharmaceutical expertise can be used to transform a drug into a medicine. There are three main categories of microbiome therapeutics: probiotics, prebiotics, and entities that alter the microbiome microenvironment (Table 3). By far the most successful of probiotic therapies is fecal microbiota transplant (FMT) for the treatment of *Clostridium difficile* infection. Traditionally, FMT involves the infusion of feces from a healthy donor into the GI tract of a patient via the rectal route, to ‘reset’ their gut microbiome composition. In *C. difficile* infection, FMT has a 92% rate of disease resolution, far outperforming traditional antibiotics. Work is increasingly underway to investigate FMT for benefit in other diseases, and formulate fecal microbiota in oral capsules to avoid transplant of whole fecal material.^4,80–82^ Prebiotics are often regarded as unregulated dietary supplements, due to their natural presence in food. There have however been several clinical trials using prebiotics for amelioration of defined diseases at specified doses. For example, 20 g of specific prebiotics daily for 6 weeks has been shown to improve gut dysbiosis in human immunodeficiency virus (HIV).^83,84^ The final category of microbiome therapeutics, entities that alter the microbiome microenvironment, is perhaps the broadest and most novel. This category includes small molecules and biopharmaceuticals that promote the growth of beneficial microbiota or dissuade the growth of pathogens.^85^ Such entities include the direct delivery of beneficial bacterial metabolites to the gut, and bacteriophages designed to lyse harmful bacteria.^86–89^

**Table 3.** Types of microbiome therapeutics

Category	Definition	Examples
Probiotics	Live microorganisms, which when administered in adequate amounts, confer a health benefit on the host.^163^	Fecal microbiota transplant for treatment of *C. difficile* infection.^80^ Oral administration of MET-3 (bacteria derived from human feces) for improvement of glucose tolerance in obese participants.^164^ Prevention of infant atopic dermatitis by *Lactobacillus* strains.^165^ Rectification of ulcerative colitis dysbiosis by administration of the commercial probiotic formulation, Symprove.^57^
Prebiotics	A substrate that is selectively utilized by host microorganisms conferring a health benefit.^166^	Improvement of HIV dysbiosis following 20 g defined prebiotics daily for 6 weeks.^83,84^ Improvement of clinical biomarkers in obese participants following administration of a daily polyphenol-prebiotic blend for 8 weeks.^167^ Histological improvement of nonalcoholic steatohepatitis following administration of oligofructose for 36 weeks.^168^
Entities altering the microbiome microenvironment	Small molecules and biopharmaceuticals that promote the growth of beneficial microbiota or dissuade the growth of pathogens.	Improvement of murine gut dysbiosis and clinical biomarkers of metabolic disease following administration of self-assembling cyclic peptides.^169^ Improvement in obese participants’ clinical biomarkers following administration of inulin-propionate ester for 24 weeks.^87^ Reduction in colorectal tumor growth following peritoneal injection of liposomal acetate for 4 weeks.^170^ Bacteriophage and bacteriocin therapy for the gut microbiome.^171^

Figure 5

**Figure 5.**
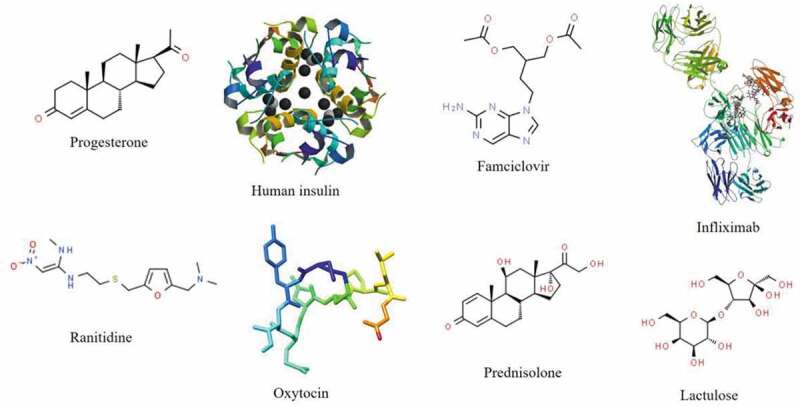
Various drugs proven to be susceptible to metabolism by gut microbiota.^10,32,33,172–177^

When formulating a medicine, there are hundreds if not thousands of factors to consider. These include route of administration, site of drug release, ease of manufacture, dosage form, and drug solubility, stability, and bioavailability.^90^ As well as being a therapeutic target, the microbiome can also be harnessed to facilitate targeted drug delivery.^91,92^ For example, fermentable carbohydrate coatings have been developed for accurate colonic drug delivery.^93,94^ Supplied with information on a drug compound, ML can quickly and accurately assess formulation options without bias.^95^ Due to the complex nature of formulation science, deep ML techniques are especially useful. Yang et al. have used deep ML to predict specific characteristics of formulations, such as dosage form disintegration and drug release.^96^ Elsewhere, deep ML has been used to predict drug solubility and oral tablet disintegration.^97,98^ Other types of ML have also shown utility in formulation design. Supervised ML, specifically random forest, has been used to predict the physical stability of solid dispersions over 6 months.^99^ Factors considered include drug molecular weight, and polymer viscosity, melting point, and topological polar surface area. The ability to predict formulation stability could save formulation scientists months of time spent iterating unstable dosage forms. Other applications include prediction of nanoparticle loading efficiency, concentration of vitamins in samples, crystallinity of drugs, and biophysical properties of monoclonal antibodies.^100–103^ As biopharmaceuticals, monoclonal antibodies share similarities with probiotics. Due to the inherent sensitivity of biopharmaceuticals, formulations should strike a balance between product efficacy, safety, stability, and manufacturability. Both proteins and live bacterial cells are susceptible to degradation in the acidic environment of the stomach, thus formulations for oral administration must adequately protect entities during gastrointestinal transit.^104^ As shown in the work by Gentiluomo et al., ML can be utilized to better understand factors that impact biotherapeutic stability.^100^ This is furthered echoed in work published by Pfizer, who developed an ML model to predict biophysical stability of antibodies using only the primary protein sequence.^105^ Perhaps in the future, the stability of probiotics in formulation could be predicted using their genomic sequence, or cell surface proteins. ML has been used to identify bacterial genes essential for life, predict antimicrobial resistance, and explore antibiotic permeation through bacterial membranes.^106–108^ In the case of bacterial membrane permeation, this could be applied to nanoparticles designed to enter bacterial cells for beneficial microbiome effects. Efficacy of formulations can also be predicted using ML. Luo et al. have illustrated the use of ML following a clinical trial, allowing prediction of changes to the gut microbiome after prebiotic consumption.^109^ This could pave the way for personalized design of formulations that maximize individual clinical outcomes. 3D printing is an additive manufacturing method with great utility in production of personalized medicines.^110–120^ The human microbiome is as unique as one’s fingerprint, thus 3D printing could be a useful technique for manufacture of personalized microbiome therapeutics.^121–127^ Such formulations could contain live microorganisms, for example oral films containing viable probiotics have been produced using inkjet printing.^128^ 3D printing of microbiome therapeutics remains in its infancy, and so ML can be well applied to efficiently optimize printing parameters; reducing the number of empirical experiments needed.^129,130^ For example, ML has been used to accurately predict the printability of medicines; this could be well applied to printing of microbiome-targeted therapeutics to optimize formulation performance.^131^ Toxicity of formulations can also be predicted by ML. For example, the skin and genital microbiomes are sensitive to the use of certain excipients.^132^ ML could predict which excipients in topical formulations may exert detrimental impacts on external commensals. Clearly, there is a lot of potential, and room for innovation, within the design of microbiome therapeutics using ML.

### Prediction of microbiome-drug interactions

Though not traditionally acknowledged, the metabolic capacity of the gut microbiome has long been equated to that of the liver.^133^ Over 180 orally administered drugs are now known to be metabolized by gut microbiota.^10,11,33,134,135^ Microbiota drug metabolism can lead to significant inter-individual pharmacokinetic variability.^136^ For example, a strain of *Eggerthella lenta* capable of inactivating the cardiac glycoside drug digoxin is estimated to inhabit the guts of 40% of the global population.^137^ Intestinal degradation of digoxin can lead to higher dose requirements of patients and make selecting a first dose difficult. Moreover, premature conversion of the Parkinson’s therapeutic levodopa by bacterial tyrosine decarboxylases in the jejunum is known to increase patients’ dose requirements.^138^ The number of drugs known to undergo microbiota metabolism may only represent the tip of the iceberg. Firstly, there are still many drugs which have not been tested for degradative susceptibility. Secondly, investigations have largely focused on bacterial interactions with drugs, disregarding contributions of viruses, fungi, protozoa, and archaea.^139,140^ Thirdly, microbiome metabolism of drugs administered by non-oral routes is under-researched. Though the human colon houses the highest density of microbiota in the body, the small intestine, skin, genital, oral, nostril, ocular, and auditory microbiomes represent unique microbial niches that could impact drugs administered by other routes.^12,141,142^ Microbiome-drug metabolism may be assessed using ML combined with biosensors, which can measure subtle alterations in pharmacokinetics *in vivo* .^143,144^ In addition to metabolism, the absorption of drugs across biological barriers may be impacted by perturbations in microbiome composition. For example, dysbiosis of the skin microbiome can affect barrier function, potentially altering the permeation of topical drugs.^145^ Moreover, dysbiosis in the colon is known to impact tight junction integrity.^57^ Whilst a database exists providing information on the impact of human genome variation on drug response (PharmGKB), comprehensive resources summarizing the impact of microbiome variation influence on drugs are lacking.^146^ The DrugBug resource curated by Sharma et al. is probably the most functional database, however it does not address pharmacokinetic concerns arising from bacterial metabolism.^51^

Due to the scale of work required to investigate microbiome effects on drugs by practical experimentation, ML offers a feasible way to predict interactions quickly and accurately. Though this work is by no means complete, there have been several progressive examples of ML use in recent years. In conjunction with practical high-throughput analysis of drug metabolism by gut bacteria, Zimmerman et al. used the clustering ML technique known as principal component analysis (PCA) to identify structural similarities of drugs susceptible to gut bacteria metabolism.^10^ Using the study’s documented 20,596 bacteria-drug interactions, PCA revealed how the presence of certain functional groups can increase the chance of metabolism. For example, drugs containing ester or amide groups are more likely to be specifically hydrolyzed by Bacteroidetes species. Other susceptible functional groups include nitro and azo moieties, which are prone to reduction by multiple anaerobic strains. This information is clearly useful for predicting untested drugs’ risk of metabolism. Another application of ML within the field of microbiome drug metabolism is work by Sharma et al.^51^ The group first constructed a database of substrates for human gut bacterial enzymes. Once assembled, the database included 1,609 substrates; 324,697 enzymes; and 491 bacterial genomes. PCA was then used to assess structural diversity of the included substrates to ensure a robust, representative model. Following this, random forest ML was employed to classify commercial drugs as being susceptible to biotransformation by specific gut microbiota enzymes. Upon analysis the prediction accuracy of the algorithm was judged to be >93%. This resource was subsequently made publicly available via the DrugBug online prediction platform.^51^ Despite being the first work of its kind, the DrugBug tool does have room for improvement. Firstly, the prediction accuracy could be improved using a larger number of drug molecules for algorithm training and testing, and by fine tweaking of algorithm parameters. Secondly, the online platform is quite slow and complicated to use. There is also no information given on the prevalence of the gut bacteria in the global population, or any clinical or pharmacokinetic implications of potential drug metabolism. DrugBug is a brilliant starting point for the ML microbiome pharmaceutics community to build on.

Whilst the microbiome can impact the actions of drugs, the same applies to the converse. A study by Maier et al. has highlighted that a significant proportion of non-antibiotic drugs impair growth of gastrointestinal microbiota.^35^ A couple of research groups have utilized this dataset of >1000 marketed drugs to build ML models that can predict anti-commensal activity of drugs untested by Maier et al.^147,148^ Such work could be highly useful in preclinical toxicity screening within the pharmaceutical industry, to predict whether new treatments could cause gut dysbiosis.

## Barriers To Overcome

Currently, the use of ML to develop microbiome therapeutics lags considerably behind other areas of science.^95,149,150^ There are several possible reasons for this, representing challenges for the field to overcome. Firstly, data describing human-microbiome-drug interactions have only begun to reach a rapid rate of discovery in recent years, with studies such as the high-throughput screening of microbiota drug metabolism by Zimmerman et al., and work from the field-leading APC Microbiome Ireland.^10,151–154^ Whilst this new abundance of information is clearly positive, to facilitate ML projects data should be collated and formatted into accessible databases. Such databases could provide information on microbiota drug metabolism, formulation parameters for microbiome therapeutics, and pharmacokinetic details for microbiota-targeted medicines. With the construction of large databases containing reliable, clean data, ML work would be easier and yield stronger predictions. Clearly, collaboration and information sharing between academic institutions and commercial corporations will be necessary for the reality of global database creation. Whilst this may be difficult in the short term, the yields will far outweigh disadvantages, likely leading to the development of new therapies for patients. Where large quantities of data are available in-house, for example historical experimental data, it may be difficult to use in ML projects.^155^ Without the foresight of storing data for potential ML use, it is likely that substantial pre-processing steps will be required.^45^ Though possible, cleaning of decades of heterogeneously formatted data can take years. There is also the barrier of skillset requirements for the adoption of ML within the field. Demand for data scientists in the United Kingdom grew by 231% in the five years preceding 2019.^156^ In addition to ML skills, it is highly beneficial for workers to also have pharmaceutics expertise, as this will facilitate optimal data mining, cleaning, and end analysis. For example, ML scientists without a pharmaceutics background may not spot anomalies or mistakes in datasets. They may also find interpretation and application of ML algorithm outputs more challenging. Training of pharmaceutical scientists in ML skills, and training of ML scientists in pharmaceutics, is thus required. Evidently, this will require substantial investment within both academic and industry settings. Finally, it is important that settings facilitate the use of ML to obtain optimal results. More so in industry, well-defined and proven systems of work will change dramatically with the widespread adoption of ML. For example, formulation scientists may spend much less time physically compounding iterations of products due to the predictive power of ML. Implementation of ML will require restructuring of procedures, in a careful and strategic manner, with full support of team members.

## Future Outlook

The explosion of big data within microbiome research in the last 20 years opens opportunities for ML like never before. Freely accessible genomic, proteomic, and metabolomic metadata facilitates commencement of ML projects with little initial investment or risk. To make the most of ML, academic and corporate institutions will need to collaborate to populate new databases with previously proprietary information, such as probiotic viability, microbiota drug metabolism, and stability studies. Whilst this will invariably raise issues around intellectual property, collaboration will offer projects not previously feasible for companies, and most importantly, will benefit patients through the development of new therapies. In order to adopt ML in a strategic, widespread, and sustainable manner, institutions will need to invest in the training of ML engineers. In the wake of a global recession instigated by the COVID-19 pandemic, institutions may find training budgets are restricted.^157–159^ Low- to middle-income countries will be especially affected, for example there is a projected 7.2% decrease in regional economic activity in Latin America compared to 4.7% in Europe and Central Asia.^160^ Though the global economic downturn will undoubtedly affect the pharmaceutical industry, it is a worthwhile investment to commit budgets to ML expertise. Once workers are trained, ML will save time and physical resources in product development. ML has the capacity to optimize and streamline the preclinical stages of microbiome therapeutics, meaning products will progress to trials faster with likely higher success rates. Major pharmaceutical companies currently showing the most activity within AI include Novartis, AstraZeneca, and Boehringer Ingelheim.^155^ Specialist AI companies, mostly working in the drug discovery space, include Exscientia, Atomwise, GNS Healthcare, and Berg. These specialist corporations typically partner with major pharma companies to unite expertise, a collaborative method of drug development that is likely to only increase in occurrence.^161^

## Concluding Remarks

Clearly, opportunities for utilizing ML in development of microbiome therapeutics are manifold. Since the early 2000s big data within microbiome research have become a reality, with genomic sequencing and metabolomic mapping providing an influx of information on the host-microbiota relationship.^12,13^ This data has provided a deepening understanding of how the microbiome can affect health, allowing the development of precision microbiome medicine. In more recent years, the impact of microbiota metabolism on drugs has come to light, and may change pharmacokinetic modeling forever.^10,162^ With the amount of data currently available, it is more important than ever to utilize systematic, accurate, and unbiased tools for analysis. ML techniques offer an accessible way to interpret metadata and use it to solve problems. Whilst a human could not feasibly recognize patterns within terabytes of information, ML can do this *and* solve complex tasks within seconds to minutes. To obtain information for ML projects, it is important to have access to reliable databases. Though several of these exist for data on drugs, microbiota, and microbiota-host dynamics, there is a distinct lack of resources providing pharmaceutical information. Curation of such databases is important for optimal utilization of ML within the field. Several challenges face institutions looking to adopt ML. Namely, cooperation with other corporations, data management, service remodeling, and lack of skillset. Whilst the necessary initial investment may be challenging for academic and industrial settings, especially considering the current global economy, actions will be worthwhile in the long term. ML is transforming almost every sector worldwide and is here to stay.
